# Nanotechnology-Based Drug Delivery Systems for Melanoma Antitumoral Therapy: A Review

**DOI:** 10.1155/2015/841817

**Published:** 2015-05-11

**Authors:** Roberta Balansin Rigon, Márcia Helena Oyafuso, Andressa Terumi Fujimura, Maíra Lima Gonçalez, Alice Haddad do Prado, Maria Palmira Daflon Gremião, Marlus Chorilli

**Affiliations:** School of Pharmaceutical Sciences, Department of Drug and Medicines, São Paulo State University, 14801-902 Araraquara, SP, Brazil

## Abstract

Melanoma (MEL) is a less common type of skin cancer, but it is more aggressive with a high mortality rate. The World Cancer Research Fund International (GLOBOCAN 2012) estimates that there were 230,000 new cases of MEL in the world in 2012. Conventional MEL treatment includes surgery and chemotherapy, but many of the chemotherapeutic agents used present undesirable properties. Drug delivery systems are an alternative strategy by which to carry antineoplastic agents. Encapsulated drugs are advantageous due to such properties as high stability, better bioavailability, controlled drug release, a long blood circulation time, selective organ or tissue distribution, a lower total required dose, and minimal toxic side effects. This review of scientific research supports applying a nanotechnology-based drug delivery system for MEL therapy.

## 1. Introduction

Malignant melanoma (MEL) are tumors that mainly affect adult and elderly patients; the highest incidence is at approximately 60 years of age [1]. However, currently, MEL recurrence has increased in young adults and can be observed in children and adolescents [2].

The World Cancer Research Fund International (GLOBOCAN 2012) estimates that there were 230,000 new cases of MEL in the world in 2012; MEL incidence rates are much higher in the White population than in the Black population, and it is uncommon in the Asian population, likely due to better protection from their skin pigment and different sun exposure habits; African and Asian societies consider fair skin beautiful [3]. In addition, rich populations have a high rate of MEL with a relatively low rate of mortality from this disease, potentially because MEL is diagnosed in early stages for this social class [4–6].

*Pathogenesis of Melanoma*. Melanocytic skin tumors include a wide variety of benign and malignant skin lesions with distinct clinical, morphological, and genetic profiles [7–9].

Melanoma describes melanocyte malignance; a melanocyte is a melanin-producing cell located in the basal layer of the epidermis [10]. When it functions normally, the melanocyte provides basic skin pigmentation and protects against UV radiation damage [11–13].

In summary, the most significant causes of MEL development are at personal history of MEL in the family, advanced age, the presence an atypical nevus, intense exposure to sunlight, sunburn during childhood [14], and chronic immunosuppression [15]; it is especially observed in posttransplant patients and patients with acquired immunodeficiency syndrome (AIDS) or a prior cancer diagnosis [11].

Genetic predisposition plays an important role in MEL development due to the relative risk of people with a family history of MEL developing this cancer, which is 2-3 times greater than in people without such a family history; several genes (CDKN2A; BRAFV600E; N-Ras codon 61; CKIT; GNAQ/GNA11; BRCA2; OCA1 and MC1R) related to this predisposition have been identified [2, 16–20].

UV radiation also has a profound influence on MEL development. Sunscreens use, which protect the skin against this radiation, does not prevent MEL development, because the UV radiation spectrum that causes erythema (UVB) and that traditional sunscreens protect against differ from the spectrum that promotes MEL (UVA). Thus, users of sunscreens are relatively unprotected from UVA radiation [11]. An alternative theory suggests that vitamin D, which inhibits the signaling pathway involved in MEL development [21] (i.e., the MAP kinase pathway that promotes cell proliferation), is synthesized upon UV radiation, and when radiation is blocked by sunscreen, vitamin D synthesis stops [22, 23].

The cutaneous MEL is manifested in different regions of the body through lesions on the head and neck and is associated with chronic sun exposure and lesions on the trunk related to the presence of numerous melanocytic nevi [24].

Almost all MEL lesions are pigmented and flat; malignant melanocytes growth is restricted to the epidermis (“MEL* in situ*”), and the cells are characterized by a relatively homogeneous brown pigmentation with slightly irregular edges [25, 26]. Over time, likely many years, these lesions present with irregular edges and pigmentation. In late stages, this neoplastic growth is vertical, and the tumor cells infiltrate through collagen fibers in the reticular dermis [27]. The subcutaneous tissue is then infiltrated by the tumor, which forms papules and nodules, and is typically confined to the lesion area [28]. Partial regression of the lesion is common, which functions through an immune mediated phenomenon that promotes malignant melanocyte elimination by cytotoxic lymphocytes [29]. However, complete MEL regression may be associated with the spread of metastasis, which is a negative, not positive prognostic sign [2, 30].

For melanocyte transformation in MEL, resistance to apoptosis is necessary [31], and MELs escape from apoptosis stimulation through overexpressing apoptosis-inhibiting genes (e.g., inhibitor of apoptosis proteins (IAPs), especially survivin) or decreasing apoptosis-inducing gene expression, which results in apoptosis dysfunction and an increased risk of metastasis [32]. The serine/threonine kinase Akt/protein kinase B and transcription factor nuclear factor-*κβ* (NF-*κβ*) participate in the cell proliferation control, apoptosis, and oncogenesis [33], and certain studies suggest that Akt activation can facilitate MEL progression by increasing cells survival through NF-*κβ* regulation with a consequent reduction in apoptosis [20].

*Classification of Melanoma and Diagnoses*. MEL is clinically classified into four main groups [34]. The first group is lentigo maligna MEL, which is characterized by an invasive tumor in the head, neck, or forearms regions [35]. Another group is superficial-spreading MEL, which is characterized by a lesion with irregular edges and pigmentation that grows laterally and slowly before promoting vertical invasion [36]. The next group is nodular MEL, which is a more aggressive type that appears in the body following high levels of sunlight exposure [37]. The final group is acral lentiginous MEL, which are pigmented lesions that appear on the palms of the hands, soles of the feet, and above the nose [38, 39]. Other classifications include amelanotic MEL, mucosal MEL, and subungual MEL [2].

For MEL diagnosis, five main characteristics of the lesion are analyzed: asymmetry, border-color, diameter, and elevation; MEL diagnoses are more accurate where dermatoscopy is used [11]. However, for many people, the first area that metastasizes is the lymph node (sentinel); the next most common site of metastasis is distant skin. The organs more frequently affected are the lungs and the liver; the central nervous system and bones can also be metastasis sites [40, 41].

The MEL stage can be determined through a complete clinical examination [42], including sonography [43] of the superficial lymph nodes and the abdomen, radiography of the thorax [44], and evaluation of serum markers, such as lactate dehydrogenase (LDH) [45], S-100-beta, sialic acid, enolase, 5-S-cyseinyldopa, 6-hydroxy-5-methyoxy-indole-2-carboxylic acid, 3,4-dihydroxy-L-phenylalanine (DOPA), L-tyrosine [46], computer tomography scan [47], magnetic resonance imaging [48], bone scintigraphy [49], and positron emission tomography, which are useful for evaluating patients with metastatic disease [50].

Moreover, an immunohistochemical technique can also be used to diagnose metastasis because antigens are expressed on malignant cells' membrane and cytoplasm surface, which can be immunohistochemically detected using antibodies that are specific to these antigens [51]. The antibodies commonly used are anti-S100, HMB-45, and MART-1 e NK1/C3.

*Conventional Therapeutic Strategies against Melanoma*. Chemoprevention can be used to avoid MEL development; chemoprevention was originally proposed by Sporn et al. (1976) [52] and refers to using synthetic or natural agents to reverse, suppress, or prevent molecular and histological premalignant lesions that occur with invasive cancer progression [20]. Reactive oxygen species play a role in MEL progression because they lead to uncontrolled overregulation and compartmentalization of melanosomes [53], a diet rich in antioxidants, particularly carotenoids and vitamins C and E, which can be used for chemoprevention [54, 55].

The conventional treatment for primary MEL is surgical; the lesion is removed, and the tissue is analyzed to determine the MEL stage, which depend on the lesion thickness and location (epidermis or dermis). The lesion is removed with a certain safety margin; however, where lesion excision is inappropriate, such as for MELs in the nasopharyngeal, sinonasal, and oral regions, radiotherapy is a way to eliminate the lesion. For patients who present risk of metastasis, the above indicated laboratory tests are also used, such as radiography of the thorax [11, 56, 57].

The conventional MEL chemotherapy treatment is performed using dacarbazine, temozolomide (dacarbazine analogue), nitrosoureas (carmustine, lomustine), vinca alkaloids (vincristine, vinblastine), platinum compounds (cisplatin, carboplatin), and taxanes (Taxol, docetaxel), but these single agents are not an improvement over dacarbazine [32, 58]. Immunotherapy has also been applied for MEL therapy; immunotherapy employs cytokines that stimulate the patient's immune system to fight cancer, such as interleukin (IL), IL-2, IL-5, IL-7, and IL-21, interferon-*α* (INF-*α*), and granulocyte macrophage colony–stimulating factor (GM-CSF) [59]. These cytokines have side effects, such as diarrhea, nausea, constipation, abdominal pain, vomiting, vitiligo, dermatitis, enterocolitis, hepatitis, toxic epidermal necrolysis, neuropathy, and endocrinopathy [11].

The benefits of therapy with interferon alfa-2b are directly related to the MEL stage [60]. However, high interferon alfa-2b doses have many side effects, such as chronic fatigue, headaches, weight loss, myelosuppression, and depression [61].

Vemurafenib and dabrafenib are BRAF inhibitors approved for use in MEL metastases that express BRAFV600E and lead to dramatic shrinkage of tumors. However, they are short-lived and resistance to treatment eventually emerges in most melanomas. In addition, treatment with BRAF inhibitors can lead to the induction of second primary cancers, including squamous cell carcinomas of the skin and new primary BRAF wild-type melanomas; other side effects are nausea, diarrhea, arthralgias, nonspecific skin rashes, fatigue, alopecia, and photosensitivity [62–64].

Tremelimumab is an antibody against the cytotoxic T lymphocyte-associated antigen 4 and is well-tolerated, but it does not offer many benefits over conventional chemotherapy [65].

Ipilimumab is a humanized antibody against CTLA-4, a negative regulatory checkpoint protein that is expressed on T cells surface after activation; the ipilimumab specifically blocks the CTLA-4 inhibitory signal, resulting in activation of T cells and tumour infiltrating lymphocytes; this is an indirect mechanism that enhances the immune response mediated by T cells. The adverse effects are colitis, dermatitis, hepatitis, endocrinopathy, and neuritis [63, 66].

Nivolumab and pembrolizumab are anti-PD-1 antibodies; PD-1, like CTLA-4, is expressed on the surface of activated T cells and has a function to turn off the T-cell response to prevent an excessive immune reaction. Anti-PD-1 antibodies may have higher response and lower toxicity rates than ipilimumab, as well as improved overall survival compared to chemotherapy [63, 66, 67].

Studies demonstrated that a combination therapy with ipilimumab and nivolumad was responsible for more adverse effects than monotherapy; on the other hand the patient's median survival was higher when patients were treated with combination therapy [66]. Therefore, several studies are being realized to evaluate the potential survival benefits of immunotherapy combination [68].

## 2. Nanotechnology-Based Drug Delivery Systems

Many active ingredients used in MEL therapy present undesirable properties and, thus, have been discarded [69]. Introducing a new active ingredient on the market takes several years of research and involves high costs. The alternative employed to circumvent these high costs and reintroduce the active ingredients that were previously discarded is the development of delivery systems that increase efficiency [70].

Drug delivery systems represent an alternative strategy to carrier antineoplastic agents. Encapsulated drug could result in advantages such as high stability, better bioavailability, controlled drug release, long circulation time in blood, selective organs or tissue distribution, a reduction of the total dose required, and minimizing the toxic side effects [71–73]. Nanotechnology-based drug delivery systems are widely used to improve the effectiveness of antineoplastic agent; the most common nanosystems are hydrogel, cyclodextrins, liquid crystalline phase, and nanoparticulate pharmaceutical drug delivery systems (NDDSs), as classified by Torchilin (2014), that include liposomes; polymeric nanoparticles; polymeric micelles; silica, gold, silver, and other metal nanoparticles; carbon nanotubes; solid lipid nanoparticles; niosomes; and dendrimers [74]. This review of scientifically based research supports the application of nanotechnology-based drug delivery system for MEL therapy.

### 2.1. Hydrogels

Polymeric systems can be classified by their physical forms such as (i) linear polymer chain in solution, (ii) physically or covalently cross-linked reversible gels, and (iii) polymer chains grafting or adsorption on the surface of micro- and nanoparticles [75]. A hydrogel is a network of polymer chains that are hydrophilic and promote the drug release through the spaces formed in the network via dissolution or disintegration of the polymeric matrix. Swelling in certain non-water-soluble polymer demonstrates a high water absorption capacity in the reticular structure (>20%) [76, 77].

An increased interest in hydrogels as a drug delivery system has been demonstrated as a result of their easy handling and similar physical properties to animal tissue, which depend on the polymer employed [78–80]. The release rate depends on the hydrogel properties, initial drug concentration, drug solubility, and drug-polymer interaction [81].

A wide variety of polymeric materials with different properties have been used to form hydrogels. The required polymer is selected based on the tissue of interest and the specific application [80, 82]. For example, poly(vinyl alcohol) tetrahydroxyborate (PVA-THB) hydrogels have shown therapeutic potential for topically treating acute and chronic wounds due to many benefits, such as controlled release, bioadhesion, and low toxicity [83, 84]. Moreover, chitosan-based hydrogels have an additional advantage as a drug delivery system because the drug can be released under various environmental stimuli; thus, these hydrogels provide an anchor for delivering therapeutic payloads to the site of action [85].

Hydrogels have been employed as a drug delivery system in MEL therapy because they may act as an intratumoral chemotherapy depot by promoting accumulation or maintenance of high intracellular levels of the chemotherapeutic agent. In recent years, a hydrogel composed of a cyclodextrin-containing linear polymer and decorated with PEG as well as transferrin was approved for commercial use in MEL therapy [86].

Hydrogels are classified as stimuli-sensitive swelling-controlled release systems because they can respond to various environmental conditions, such as pH, the surrounding fluid ionic strength, temperature, an applied electrical or magnetic field, or glucose level changes. These changes promote altered network structure, swelling, mechanical strength and permeability [83]. Thus, hydrogels may be used to improve drug delivery [87–90]. However, little evidence supports using hydrogels for topical treatment of MEL.

Certain studies suggest using topical hydrogels; topical ibuprofen-releasing hydrogels promote lower metastatic spread of primary MEL through significantly lower tumor necrosis factor (TNF)-*α* levels, which is the major proinflammatory cytokine that induces MEL cell migrations [91].

Injectable hydrogels have been widely explored for cancer therapy [85, 92]. Interleukin-2 was given as pulse in cancer immunotherapy because it is a potent immunomodulator that can induce antitumor activity [93]. Recombinant human interleukin-2 (rhIL-2) loaded,* in situ* gelling, and physically cross-linked dextran hydrogels slowly release rhIL-2 and maintain the rhIL-2 protein in an intact form that is both biologically and therapeutically active, which greatly enhances the clinical applicability of rhIL-2 immunotherapy [94].

Subcutaneous injection of a doxorubicin-loaded hydrogel composed of sugar beet pectin (SBP) associated with biodegradable gelatin (SBP/gelatin) successfully suppressed mouse MEL B16F1 cell tumor growth in nude mice [95].

The human MEL cell line Me665/2/21 derived from a cutaneous metastasis was treated for 48 h with a cisplatin-loaded hydrogel and it showed similar and, in certain cases, higher cytotoxic activity towards the MEL cell line compared with free cisplatin at the same concentration [79].

A novel system for incorporating paclitaxel has been investigated to lower toxicity and improve efficacy [96]. Tumor activity upon using a paclitaxel (PTX) loaded hydrogel composed of a pH- and temperature-sensitive block copolymer, the poly(*ɛ*-caprolactone-co-lactide)–poly(ethylene glycol)–poly(*ɛ*-caprolactone-co-lactide) (PCLA–PEG–PCLA) block copolymer, was analyzed* in vivo* using B16F10 MEL cells. After 2 weeks of subcutaneously injecting the B16F10 MEL cells into male mice, the tumors were allowed to grow, and the results demonstrate that saline-treated mice produced a tumor volume of approximately 17 cm^3^, while the PTX-treated mice tumors were smaller than 7 cm^3^, which demonstrates that a PTX-loaded block copolymer hydrogel can effectively suppress tumor development [97].

### 2.2. Liposomes and Micelles

Recently, research has described the importance of lipids in drug carrier systems such as liposomes [98]. According to Fahy and coworkers (2005) [99], lipids are hydrophobic molecules that are soluble in organic solvents. However, certain lipids have amphiphilic characteristics due to both hydrophilic and hydrophobic segments [100]. These compounds can form carrier systems, such as micelles and liposomes, because they can self-assemble in the presence of water [101]. Micelles are formed when amphiphilic components concentration exceeds a certain threshold concentration. The micelles' size and shape depend on pH, temperature, constituent geometry, and intermolecular interactions [98, 99, 102, 103].

**Figure 1:**
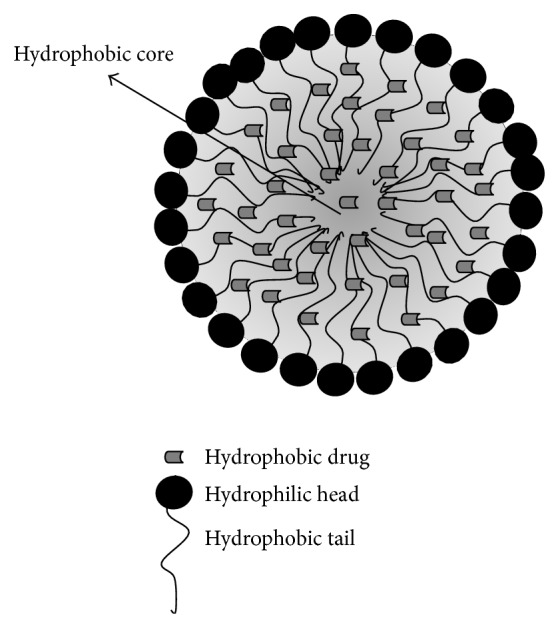
Micelle with hydrophobic compounds.

**Figure 2:**
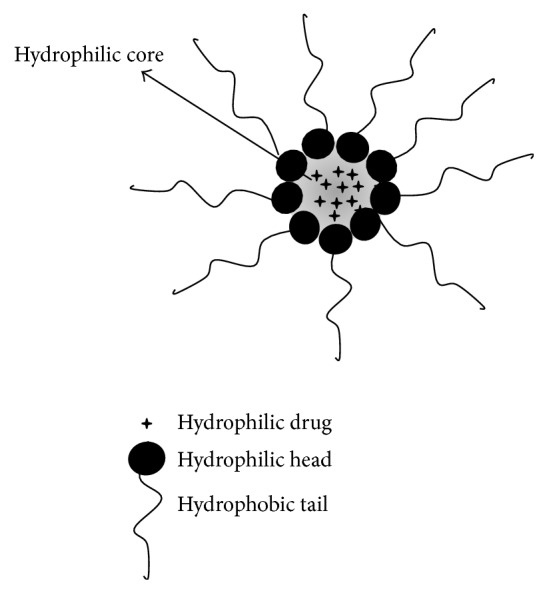
Micelle with hydrophilic compounds.

**Figure 3:**
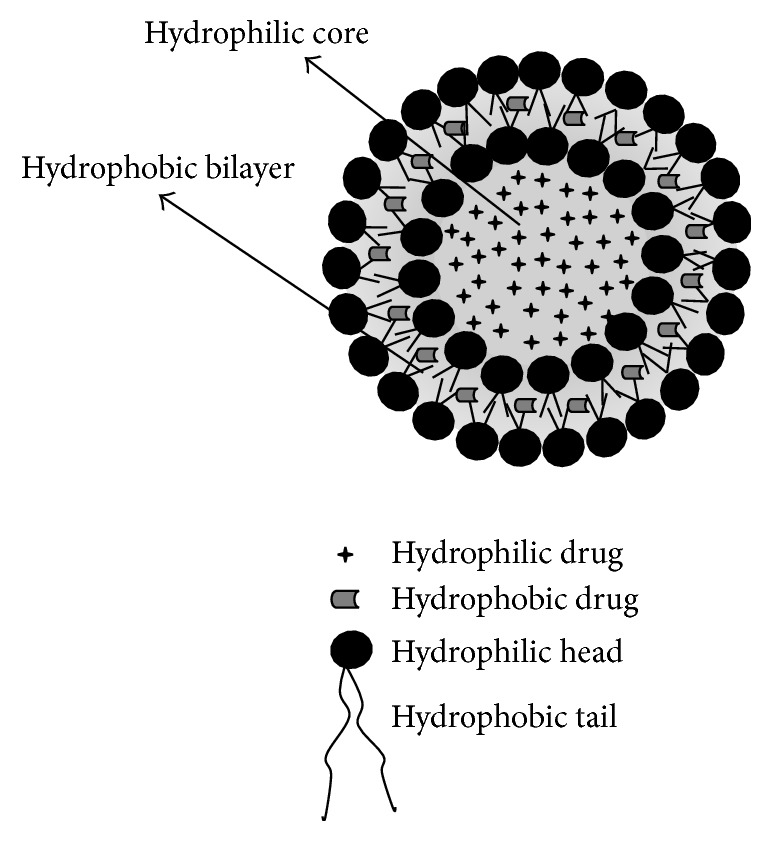
Liposome encapsulated hydrophobic and hydrophilic compounds.

The liposomes are microscopic spherical nanostructured with a well-defined shape and size, which varies from 10 nm to several micrometers, depending on the technique used to create them [104]. These vesicles are formed by an external phase with double phospholipid membranes and an internal phase formed by an aqueous medium [105]. These components provide an amphiphilic character due to the organized double phospholipid layer that surrounds the aqueous compartment. Thus, they can encapsulate both hydrophobic and hydrophilic compounds [106, 107]. Figures 1, 2, and 3 schematically show the micelles and liposomes structures.

Liposomes have high versatility because they can be modified based on pharmacological and pharmaceutical needs. Thus, the size, surface, lamellarity, lipid composition, volume, and inner aqueous medium composition can be modified in these vesicles [108].

Liposomes can be formed with natural lipids, such as sphingomyelins, as well as lecithins and synthetic lipids, such as dimyristoyl, distearoyl, dipalmitoyl, and dioleoyl [109]. Currently, there are several methods to obtain liposomes. Under certain conditions, they can undergo spontaneous rearrangement and be derived from preformed micelles by changing the solution or applying external energy, such as by extrusion through filter membranes, sonication, or agitation [98, 110, 111].

The extrusion technique forces a lipid suspension to pass through a polycarbonate membrane with a well-defined pore size [112]. This method can produce vesicles with a diameter near the membrane pore size used to prepare the liposomes. Over time, studies have shown several advantages from this technique; for example, the average size of the vesicles formed is reproducible due to the physical process involved in liposome formation, residual organic solvent removal at the end of the technique is unnecessary, and a large variety of lipids can be used to prepare the vesicles [111, 113, 114].

For the sonication technique, liposomes are prepared using a sonicator to mix the lipid suspension. The pressure exerted by the sonicator stirring causes a decrease in the larger vesicles sizes. Thus, the stirring time is decisive for liposome size formed. The main advantage of this technique is less time in liposome preparation [111, 115].

Liposomes have attracted the attention of the scientific community due to their high versatility. Liposomes have greater therapeutic efficacy than conventional pharmaceutical system because they promote slow drug release at the target site [107, 116, 117]. Furthermore, liposomes are less toxic, nonimmunogenic, and biocompatible with organic tissues. They can decrease systemic toxicity and improve drug efficacy, especially for antibiotics, antifungals, and anticancer drugs [107, 118, 119]. Thus, using liposomes as a delivery system for chemotherapeutic agents offers great prospects for cancer treatment [108].

Wolf et al. (2000) [120] incorporated the DNA repair enzyme, T4 endonuclease V, into liposomes composed of phosphatidylcholine, phosphatidylethanolamine, oleic acid, and cholesteryl hemisuccinate (2 : 2 : 1 : 5 molar ratio) and applied it to human patients with a previous history of skin cancer after ultraviolet exposure. These liposomes were developed by encapsulating a purified recombinant T4 endonuclease V. The researchers observed that the enzyme was present in skin cells, which, in the skin, tends to improve DNA repair. Moreover, they reported that the T4 endonuclease V liposome prevented ultraviolet-induced upregulation of tumor necrosis factor-alpha and interleukin-10 mRNAs as well as interleukin-10 protein.

Another study using T4 endonuclease V in liposomes was conducted by Yarosh et al. (2001) [121]. They observed 30 patients with xeroderma pigmentosum in a double-blind study. The patients were randomly assigned to use either the T4N5 liposome or a placebo liposome lotion, daily for 1 year. To produce the T4 endonuclease V liposome lotion, they used 1 mg/L T4 endonuclease V encapsulated in liposomes in a 1% hydrogel lotion. The placebo lotion was prepared with the same liposomes in a 1% hydrogel solution but without the enzyme T4 endonuclease V. Patients with xeroderma pigmentosum have a genetic error in a DNA repair enzyme. Thus, the incidence of skin cancer in these patients is more frequent than in other people. The researchers noted a decrease in the xeroderma pigmentosum incidence rate and skin cancer in the groups treated with the T4 endonuclease V liposome. Furthermore, no significant adverse effects were reported.

Pierre et al. (2001) [122] proposed a topical delivery system for 5-aminolevulinic acid (5-ALA) based on liposomes with a similar composition to the stratum corneum to treat skin cancer. They prepared these liposomes using a reverse phase evaporation technique and the following components: ceramide, cholesterol, palmitic acid, cholesteryl sulfate, and *α*-tocopherol. 5-ALA is used in photodynamic, which had been shown effective in topical treatment for a variety of skin diseases. 5-ALA liposomal delivery system targeted and delivered 5 ALA to skin layers (viable epidermis and dermis) compared with the aqueous solutions typically applied in a 5-ALA-PDT clinical procedure. Thus, liposomes were a suitable delivery system for 5 ALA.

Chen et al. (2012) [123] developed a transdermal drug delivery system for curcumin-loaded liposomes and investigated* in vitro* skin permeation and the antineoplastic effect* in vivo*. Soybean phospholipids, hydrogenated soybean phospholipids, and egg yolk phospholipids were used to obtain the liposomes. Curcumin-loaded liposomes composed of soybean phospholipids promoted greater* in vitro* drug permeation. Moreover, the liposomes were effective against MEL and the liposomes composed of soybean phospholipids showed a higher capacity to inhibit MEL cells growth.

Nobayashi et al. (2002) [124] evaluated the efficiency of cationic multilamellar liposome-mediated gene transfer in murine MEL cell lines and an experimental gene therapy for subcutaneous MEL. They used B16F10, which is a murine MEL cell line and cationic liposomes composed of* N*-(*α*-trimethylammonioacetyl)-didodecyl-D-glutamate chloride (TMAG), dilauroyl phosphatidylcholine (DLPC), and dioleoyl phosphatidylethanolamine (DOPE) at molar ratio 1 : 2 : 2. They observed that repeated exposure to liposomes increased the transduction efficiency in murine MEL cells and experimental subcutaneous MEL tissue; thus, the therapy was effective for the intended purpose.

Liu and colleagues (2013) [125] developed liposomes with quercetin to improve its delivery into human skin and evaluate the potential anti-UVB effect. The liposomes were composed of soybean phosphatidylcholine, cholesterol, tween 80, and span 20. The researchers prepared liposomes with high entrapment efficiencies and a prolonged drug release. The group yielded good results; the quercetin liposomes enhanced cell viability compared with a quercetin solution in UVB-irradiated HaCaT cells, the reactive oxygen species levels decreased, the edema and inflammation were alleviated and 3.8-fold more quercetin liposomes permeated the skin compared with quercetin solution.

### 2.3. Cyclodextrins

Cyclodextrins (CDs) are a family of natural cyclic oligosaccharides with *α*-(1-4) linked glucopyranose subunits bonds [126–128]. They are produced from starch via enzymatic conversion using cyclodextrin glycosyl transferases (CGTases) [129, 130]. CDs have received more attention as a pharmaceutical excipient, because they can form drug complexes [130–132]. Furthermore, CDs are biocompatible and can be used to reduce* in vitro* and* in vivo* toxicity and the delivery profile can be modulated with great flexibility by changing the guest components [133].

*β*-cyclodextrin (*β*CD) is a CD that comprises seven *α*-(1,4)-linked *α*-d-glucopyranose units and is used extensively due to its ready availability and because its cavity size is suitable for a varied drug range [131]. Many hydrophilic, hydrophobic, and ionic CD derivatives have been developed to increase CDs versatility and decrease undesirable drug properties [134, 135].

Recent studies have demonstrated that CDs are efficient drug delivery systems for targeting cancer cells [136–138]. A complex formed between CD and a gemini surfactant (CDgemini) was used to carry curcumin analogue, and the cytotoxic effect of this system in MEL cells was analyzed. The results indicate that the drug-loaded CDgemini showed higher caspase 3/7 activity levels compared with drugs dissolved in DMSO, which enhance their ability to trigger apoptosis. Further, the researchers demonstrated that this treatment was more specific for MEL cells than for healthy keratinocytes [139].

In general, the pH surrounding tumor tissues tends to be more acidic (i.e., ~ 5.5 to 6.5) than normal tissue (i.e., 7.4) [140, 141]. Thus, pH-triggered drug release systems are promising for intracellular delivery of anticancer drugs [142]. Certain substances exhibit pH-sensitive host-guest interactions with cyclodextrin and may be used as pH-triggered drug release systems [143]. He and coworkers (2013) [144] synthesized a pH-responsive material through acetonating *α*-CD for PTX delivery. Results from* in vitro* drug release studies show a more rapid release profile in pH 5.0 buffer comparison with physiological conditions (pH 7.4). Moreover, this system can be effectively internalized by tumor cells; it demonstrates a superior cytotoxic activity and a longer incubation time results in higher efficiency. In addition, treatment with PTX loaded pH-sensitive *α*-CD inhibited tumor growth even at the lower PTX dose (1.1 mg/kg) [144].

Polypseudorotaxanes are inclusion complexes formed between cyclodextrins and linear macromolecules such as polymers [145]. Doxorubicin (DOX) loaded polypseudorotaxanes were developed by Chang and colleagues (2013) [146] and* in vitro* antitumor studies including cellular uptake and inhibition efficiency were analyzed for B16 MEL cells. The results indicate that doxorubicin-loaded polypseudorotaxanes inhibited MEL cells proliferation. The loaded doxorubicin showed slower endocytosis than doxorubicin hydrochloride, perhaps due to the larger system size. The cellular uptake of loaded doxorubicin was greater upon increasing the incubation time. Polypseudorotaxanes may be a promising carrier for DOX as antitumor MEL therapy.

4-Hydroxynonenal (4-HNE) is the end product of lipid peroxidation, which has been broadly used to inducer oxidative stress, and it produces a cytotoxic effect in cancer cells [147, 148]. The 4-HNE inclusion complex with the derivative *β*CD (PACM-*β*CD) was developed by Pizzimenti and coworkers (2013) to enhance 4-HNE stability [149]. The results demonstrate that the inclusion complex HNE/PACM-*β*CD was stable and significantly reduced more viable cells among the several cell lines tested, including human MEL A375 cells, than untreated control cells and cells treated with 10 *μ*M free HNE.

Disrupting the lipid rafts' integrity, which are plasma membrane microdomains rich in cholesterol, may modify tumorigenic processes by altering the functionality of CD44, which is a cell surface receptor involved in cell migration and tumor metastasis [150, 151]. Murai and colleagues (2011) [152] showed that cholesterol reduction might be effective for preventing and treating malignant tumors progressions. Methyl-*β*-cyclodextrin (M*β*CD) forms soluble inclusion complexes with cholesterol and depletes cholesterol in plasma membranes [153]. A study conducted by Onodera et al. (2013) [154] investigated the potential of M*β*CD to cause apoptotic cell-death in a highly pigmented human MEL cell line. The results demonstrate that M*β*CD induced apoptosis through cholesterol depletion in lipid rafts, which activated caspase-3/7 and promoted cancer cell apoptosis. Thus, M*β*CD provides a potential strategy for treating MEL via lipid rafts modulation.

Mazzaglia and coworkers (2013) [155] developed an amphiphilic cyclodextrin (ACD) system for incorporating porphyrin derivatives to improve their water solubility and their selectivity towards MEL cells. The complexes formed showed higher cytotoxic activity in MEL cells than the free porphyrin derivative in water; thus, apoptotic cell death was observed at lower concentrations, and both cell proliferation and changes in cellular morphology were inhibited.

Mistletoe extract is often used in complementary cancer therapy [156]; it has been shown to stimulate cytokine production, modify intracellular protein synthesis, induce cell necrosis, and inhibit tumor colonization [157]. Strüh et al. (2013) [158] solubilized mistletoe triterpenoids with cyclodextrins and observed lower tumor growth, tumor necrosis, apoptotic cells, and prolonged survival in mice. These results indicate that solubilized mistletoe triterpenoids enhanced the antitumor effect of mistletoe extract.

Betulin (BET) is found in* Betula* sp. and has been used to treat skin diseases due to its therapeutic properties, including antitumor activity [159, 160]. Complexes formed between BET and a novel CD derivative, octakis-[6-deoxy-6-(2-sulfanyl ethanesulfonate)]-*γ*-CD (GCDG) were developed, and* in vitro* and* in vivo* experimental animal model experiments were conducted to verify antineoplasic activity in system. The results showed that BET complexation with CD improved BET solubility, which was an important property for enhancing BET antitumor activity. Moreover, BET promoted a lower MEL size, which was attributed to its antiangiogenic effect [160].

Interleukin-2 (IL-2) promotes immune recognition of MEL, while sparing normal cells [161]. However, secretion of certain immunosuppressive factors, such as TGF-*β* (transforming growth factor-*β*), can decrease ability of the immune system to identify the tumor as being composed of foreign cells [162]. A system composed of methacrylate-f-CD to solubilize the TGF-*β* inhibitor and liposomes loaded with a biodegradable crosslinking polymer and IL-2 cytokine was developed to sustain cytokines release to the tumor microenvironment and induces antitumor immune responses in a B16/B6 mouse. The results show that the TGF-*β* inhibitor and IL-2 reduced tumor growth. Furthermore, the natural killer cells' activity increased [163].

Cancer photodynamic therapy (PDT) combines a photosensitizer or photosensitizing drug with a specific type of light source to treat cancers [164]. A nontoxic carrier was prepared using 2-hydroxypropyl-cyclodextrins (hpCDs) and metallocomplex* mes*o-tetrakis(4-sulfonatophenyl)porphyrin (ZnTPPS4) as the photosensitizer. The results demonstrate that low irradiation doses do not promote a substantial damaging effect on MEL cells, whereas higher irradiation doses induce cell death. Cell apoptosis or tissue necrosis depends on the radiation intensity. ZnTPPS4 complexation was an efficient sensitizer in human MEL cells [165].

### 2.4. Liquid Crystalline Phases

Pharmaceutical companies have shown an interest in developing nanostructured systems, such as liquid crystals, which have advantages that are mainly related to controlled drug release, and protect the active ingredients from thermal degradation or photobleaching [166, 167].

Liquid crystalline systems can compartmentalize drugs in the inner phase droplets, which have different physicochemical properties than the dispersing medium, and induce changes in the biological properties of the incorporated substances [168, 169].

Lehmann described an intermediate state in the thermal transformation from solid to liquid, which became known as liquid crystals (CLs) [170–172].

Liquid crystals are classified as lyotropic and thermotropic. When these systems are formed through adding solvents, they are lyotropic; thermotropic formation is temperature-dependent. As the surfactant concentration changes occur, different liquid-crystalline forms can be generated, such as lamellar, hexagonal (hexasomes), and cubic (cubosomes) forms. The lamellar phase is formed by parallel, planar layers of surfactant bilayers separated by a solvent layer, which form a one-dimensional network. Beginning in the hexagonal phase, aggregates are formed through an arrangement of long cylinders that form two-dimensional structures. In the cubic phase systems, the molecules are arranged in a three-dimensional system that consists of two corresponding water channel networks surrounded by lipid bilayers or surfactant [169].

Polarized light microscopy is an important tool to identify and classify liquid crystalline materials. Photomicrographs are used to demonstrate the observed textures, typically using polarized light [173]. Under polarized light plane, the sample is anisotropic if it can divert the plane of incident light that is isotropic and does not deflect light. The lamellar and hexagonal mesophases are anisotropic, while the cubic mesophase is isotropic [169, 174].

**Figure 4:**
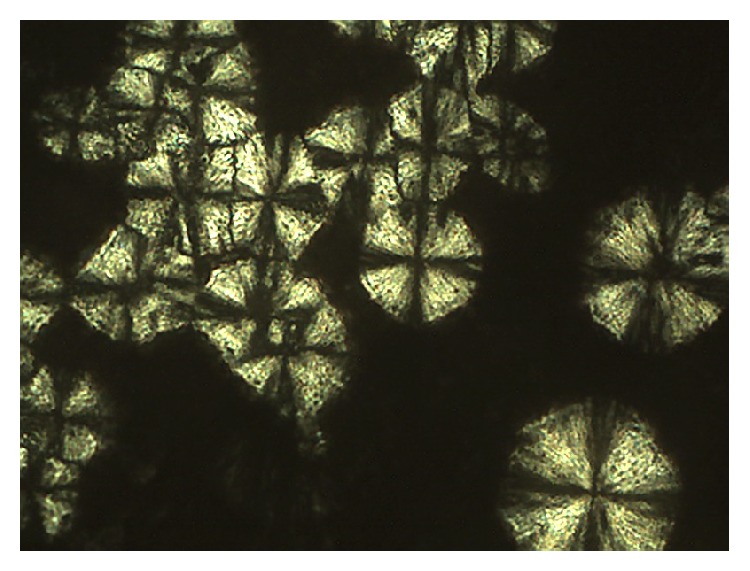
Polarized light microscopy of the lamellar phase (anisotropic system).

**Figure 5:**
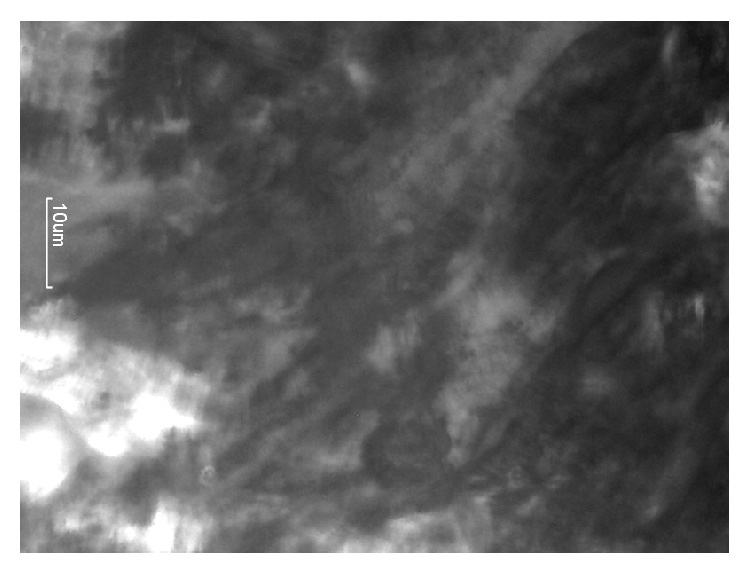
Polarized light microscopy of the hexagonal phase (anisotropic system).

Figures 4 and 5 show microscopy systems in lamellar and hexagonal phases, respectively.

Liquid crystals have increasingly been used as delivery systems; Bitan-Cherbakovsky and colleagues (2013) [175] evaluated the release of gallic acid in cancer treatments. Liquid crystalline systems were studied as a dermal delivery system with ascorbyl palmitate to prevent skin aging [176].

Cubosomes present potential utility as a drug delivery system in skin cancer therapy, such as for MEL, due to their bioadhesion properties and enhancer penetration [177]. Bei and coworkers (2010) formulated dacarbazine-loaded cubosomes composed of glycerol monooleate RYLO MG 19 (GMO), poloxamer 407 (F127), phosphate buffer saline (PBS), and DTIC (5-(3, 3-dimethyl-1-triazeno) imidazole-4-carboxamide) and characterized their physicochemical properties. Currently, dacarbazine is a first-line chemotherapy agent against MEL. Due to the material's bioadhesion properties, it presents a potential for use in MEL therapy [178].

5-FC phytanyl (5-FCPhy) is an amphiphile prodrug, carried in a lyotropic liquid crystalline system, and its* in vivo* efficacy as a chemotherapy agent against breast cancer has been investigated. The results show that the 5-FCPhy-loaded lyotropic liquid crystalline system reduced tumor size in a dose-dependent manner; the smallest average tumor volumes were observed with highest 5-FCPhy doses. Thus, a 5-FCPhy-loaded lyotropic liquid crystalline system may be used as a controlled drug delivery system for chemotherapeutic treatments such as for MEL [179].

von Eckardstein et al. (2005) developed an intracavitary carrier system composed of cubosomes that encapsuled carboplatin and paclitaxel; the release kinetics, the antitumor activity against glioma, and the prolonged survival were analyzed. The results show a significantly smaller tumor in animals treated with paclitaxel/carboplastin compared with the control group although survival did not differ among the groups studied. Both the drugs carried in the crystalline cubic phases presented cytotoxic activity in tumor cells, which indicates that they play an important role in cancer therapy [180]. The same researchers clinically observed 12 patients with a recurrent glioblastoma multiforme, who received an intracavitary application of paclitaxel and carboplatin cubosomes in different doses. The results indicate that this system is feasible and safe [181].

Many studies show the advantages of liquid crystals as a drug delivery system. However, most studies conducted using liquid crystals as a chemotherapy drug delivery systems remain at an early development stage. Several studies have been executed to characterize certain systems without efficacy trials [178, 182–186]. However, more studies are necessary to better understand the role of liquid crystals as a drug delivery system in MEL therapy.

### 2.5. Nanoparticles

The Food and Drug Administration (FDA) defines a nanoparticle as any material with a dimensional range of approximately 1 to 100 nm or end products with a dimension up to 1 *μ*m that exhibit properties or biological phenomena (chemical, physical, and biological effects) [187–192]. Nanotechnology-based drug delivery systems have gained scientific notoriety due to variety of applications and many benefits; these systems may include polymeric and lipid-based nanoparticles.

In 1996, Müller and Lucks introduced the term solid lipid nanoparticle (SLN) to patent a manufacturing process using high pressure homogenization [193]. SLNs are the first generation of lipid nanoparticles (LN), which can be constructed by only using solid lipids (i.e., lipids that are solid at room temperature) [194].

**Figure 6:**
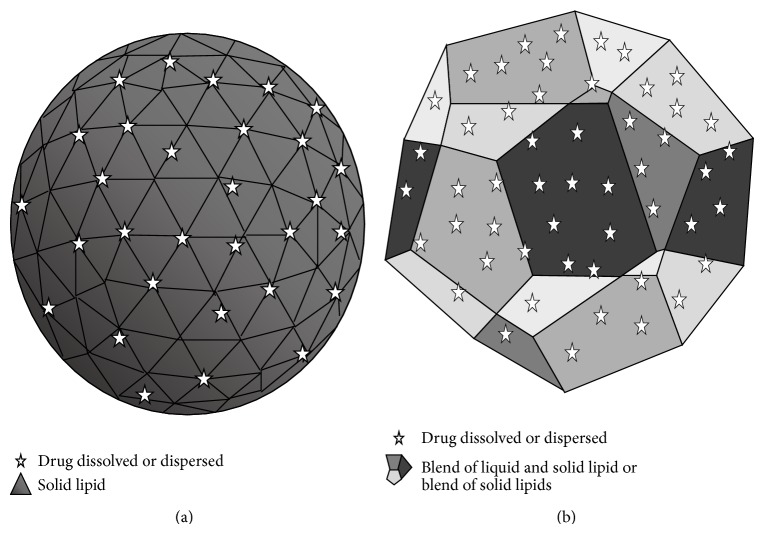
The image shows an SLN-organized lipid matrix composed of only solid lipids (a) and imperfections in the crystal lattice (b) on NLC or SLN that are composed of multiple solid lipid components with distinct structural features that are distorted upon forming a perfect crystal.

Subsequent modifications to SLNs have been described, which are nanostructured lipid carriers (NLC) and are the second generation of LN [194]. Both SLN and NLC are constructed from lipid solid. However, they can be distinguished by their internal structures. The internal SLN structures only have solid lipids and NLCs are constructed using a blend of solid and liquid lipids, which produces imperfections in the crystal lattice [195, 196], as shown in Figure 6. These imperfections have also been observed for SLNs because SLNs that contain multiple solid lipid components with distinct structural features may improve the drug entrapment efficiency [195, 197, 198].

In addition to LN, polymeric nanoparticles (PN) may be constructed from organic polymers or inorganic materials, such as silica [199]. Polymers or lipids form solid NP nuclei, which promotes more stable systems, sustained drug release, and a uniform particle size distribution [200].

**Figure 7:**
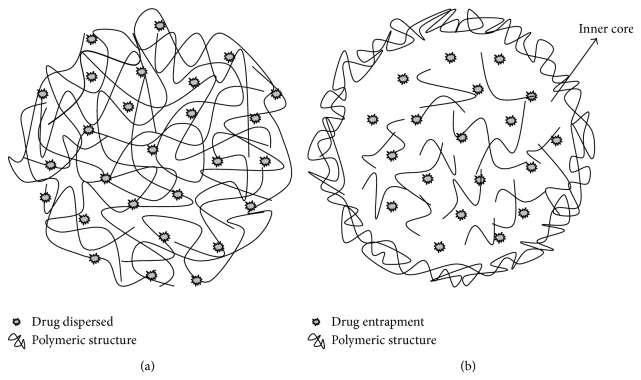
Polymeric nanoparticles schematics: nanospheres (a) and nanocapsules (b).

PN can be referred to as nanocapsules or nanospheres depending on their composition [166], as shown in Figure 7. The presence of oil promotes a vesicular structure in nanocapsules that forms reservoir-based drug delivery systems [201, 202], while nanospheres form matrix organized polymeric chains in the absence of oil [203, 204].

Drugs are entrapped in PN throughmixing the drug and polymer solution. Drug compounds are physically entrapped in the nanoparticle through polymer self-assembly [200]. PNs using different encapsulation mechanisms, such as dissolving it, disperse it or chemically adsorbs it in the constituents of the polymer matrix [166, 205, 206].

Both types of nanoparticles (lipid and polymeric nanoparticles) can be used as a drug delivery system with antitumor properties in MEL therapy.

Identifying tumor microenvironment properties is critically important for accumulating the most nanoparticles at the site of action, which decreased drug toxicity and adverse effects. Pathological systems' metabolism, cell morphology, and microenvironment have peculiar characteristics [207]. Through knowing these characteristics, specific biomarkers (antibodies, aptamers, peptides, and small molecules) can be identified, and molecules can be attached to the nanoparticles surface for successful targeted drug delivery to the site of action [208, 209].

Nonspecific interactions may appear in addition to specific biomarkers such as van der Waals bonds and electrostatic and steric affinities that can be used to predict the propensity for nanoparticle adhesion and uptake [210]. Thus, the particular nanoparticles structural components (lipids, surfactants, and polymers: Table 1) may improve drug targeting to the tumor tissue [198, 211], prevent opsonization and a consequent decrease in nanoparticles degradation by the immune system [212–214], improve the interactions between the surface nanoparticles and tumor cell membrane [215–217], alter the normal function of organelles, and induce apoptosis, which would increase tissue-specific cytotoxicity in cancer cells [197, 218, 219].

#### 2.5.1. Benefits of Using Nanoparticles for MEL-Targeted Drug Delivery

Recent studies have shown improved SLN uptake and accumulation in tumor tissue [220], due to physiological tumor tissue characteristics, such as abnormalities and a dysfunctional tumor vasculature, which allow SLN in the range 30–100 nm to easily permeate the tumor. Moreover, higher SLN concentrations are maintained in the tumor for long periods of time due to low venous return and lymphatic drainage [221–223].

Xu and coworkers (2009) development a docetaxel-loaded SLN composed by egg phosphatidylcholine, dioleoylphosphatidylethanolamine, trimyristin, and lactobionic acid that showed 2.4-fold greater accumulation in tumors compared with the nonencapsulated drug 6 hours after intravenous administration. Galactosylation of the nanoparticle surfaces enhanced the cellular uptake of docetaxel and promoted passive targeting of the drug into the tumor cell, which reduced systemic toxicity [224]. Higher cationic nanoparticle uptake in HeLa cells compared with anionic nanoparticles was observed [225], which demonstrates that nanoparticle uptake is influenced by the nanoparticle surface molecules [226, 227].

Guo et al. (2010) investigated the antitumor effects of resveratrol (RES) bovine serum albumin nanoparticles. The results showed that the concentration of RES was greatly increased in the target tissue when the RES-loaded nanoparticle was injected. High levels of RES were observed in bloodstream for long periods of time after the RES suspension was administered (nonencapsulated RES), which illustrated incomplete RES distribution. Moreover, the results show that RES-loaded nanoparticles promoted greater tumor growth inhibition [228]. Teskač and Kristl (2010) showed that where NLC (Compritol 888ATO and Phospolipon 80H as the oil phase and Lutrol as the steric stabilizer) was used to incorporate RES, it crossed the cell membrane, was delivered throughout the cytosol, and was located in the perinuclear region without inducing cytotoxicity. They found that RES solubility, stability, and intracellular release were also enhanced in the RES-loaded nanoparticle [229].

The drug release profile was modulated using drug-loaded LN. A recent study demonstrates that the camptothecin release rate can be modified by changing lipid nanoparticle inner phases. The SLN composed of precirol as the solid lipid showed the most sustained release (45% of the total drug was released after 30 hours) while the NLC composed of precirol as the solid lipid and squalene as the liquid lipid showed more rapid release (65% of the total drug was released after 30 hours). Drug mobility decreased when solid or crystalline substances were incorporated into the nanoparticles, which decreased the drug levels released as a function of time. Greater inhibition of MEL cell proliferation was observed when the cells were treated with nanoparticles, which may be because MEL cells exhibit excellent uptake endocytosis [230].

Docetaxel-loaded NLC (DTX-NLC) composed of stearic acid, glyceryl monostearate, soya lecithin, and oleic acid showed as sustained-release drug profile (77% of the total drug after 24 hours), while Duopafei (docetaxel injection provided by Qilu Pharmaceutical Co., Ltd. in China) showed 100% release after 24 hours. In addition, DTX-NLC showed greater cytotoxicity against MEL cells compared with Duopafei through enhanced apoptosis. Moreover, DTX-NLC showed low cytotoxicity for healthy cells because the drug is only released after endocytosis by a target cell [231].

Camptothecin was encapsulated into NLC, which was composed of cetyl palmitate, coconut oil, and Myverol associated with a quantum dot (metallic compounds at the core of the semiconductor NLC) as oil phase and Pluronic 68 solution as water phase. Camptothecin-loaded NLC presented superior cytotoxicity against MEL cells and the greater cell uptake compared with other carriers. Cellular endocytosis was essential for cell viability and the quantum dots showed a minimal capacity to influence proliferation. In addition, camptothecin accumulation in the MEL increased by approximately 6.4-fold following administration of the camptothecin-loaded nanoparticle.* In vivo* real-time monitoring showed that a camptothecin-loaded nanoparticle associated with a quantum dot was strongly localized at tumors with a persistent signal for 24 hours. The drug-loaded NLC was directed using quantum dot to efficiently transmit sustained tumor bioimaging, in addition to promoting drug release. This system offers the potential for diagnosing or monitoring evolution of the tumor through bioimaging and for drug delivery through nanocarrier [232].

Multiple synthetic and natural biodegradable polymers may be used in antitumor drug delivery systems, such as polyesters (e.g., polylactic acid, PLA), polyamino acids (e.g., polyaspartic acid), and polyoxypropylenes (e.g., poloxamers) [233].

Interleukin-2 was delivered by a polymeric nanoparticle composed of a low molecular weight polyethylenimine linked by *β*-cyclodextrin and conjugated with folate; this molecule was analyzed as a potential MEL antitumor therapy. High levels of tumor suppression were observed after peritumoral injection of the polymeric nanoparticle with interleukin-2, which prolonged survival in mice; thus, it is a promising gene-based therapeutic strategy for MEL. The antitumor effect can be attributed mainly to activation, proliferation, and infiltration of effector T cells and NK cells into the tumor; the therapeutic efficiency was dose-dependent and presented low cytotoxicity [234].

Polymeric nanoparticle using polylactic-co-glycolic acid (PLGA) as a polymer to incorporate coumarin increased the cellular uptake rate 2-fold versus nonencapsulated coumarin. In addition, molecular signals for mRNA expression were used to demonstrate that the coumarin-loaded nanoparticle downregulated cyclin-D1, proliferating cell nuclear antigen (PCNA), survivin, and Stat-3, and it upregulated p53 and caspase-3, promoting enhanced apoptosis of MEL tumor cells compared with nonencapsulated coumarin [235].

As a drug delivery system for apigenin, PLGA-PN promotes faster mobility and site-specific activity in MEL in addition to efficiently preserving apigenin photodegradation. The results also showed increased free radical accumulation and antioxidant enzymes depletion inside tumor cells, which exacerbated DNA damage and results in apoptosis through mitochondrial dysfunction [236].

A polymer-based delivery vehicle for cisplatin composed of chitosan and carboxymethylcellulose showed enhanced cytotoxicity (approximately 10-fold greater) in MEL tumor cells compared with nonencapsulated cisplatin. Further, rapid intracellular drug release was observed upon endocytosis of this system by a tumor cell, and only high-density NPs and positively charged-surfaces were capable of releasing cisplatin into MEL. Moreover, it decreased drug loss during blood circulation [237].

Superparamagnetic iron oxide nanoparticles consist of a carboxydextran shell and show increased uptake in human mesenchymal stem cells; the nanoparticle uptake efficiency was related to a higher density of carboxyl groups on the nanoparticle surface [238].

## 3. Advantages and Disadvantages of Nanocarrier Systems

This paper describes umpteen benefits to use nanocarriers system to vehiculate drugs used in melanoma therapy. But, to choose the better system type, it is also necessary to analyze the disadvantage of each system.  Table 2 describes main advantages and disadvantages of each system.

## 4. Nanocarriers Application in Animal Model or Clinical Studies

Particular nanocarrier structural components were previously described for improving drug targeting to the tumor tissue (Table 1). Now, some animal models studies or clinical efficacy will be portrayed.

A study realized by Shi and coworkers (2014) demonstrated that microRNA-34a and paclitaxel-loaded functional cationic solid lipid nanoparticles presented a synergistic anticancer efficacy.* In vivo* test was conducted and this system was much more potent inhibitor of B16F10-bearing tumor growth and can eliminate melanoma metastasized to the lungs cells compared to single drug-loaded SLN [273].

C57BL/6 mice were inoculated subcutaneously with B16-F10 melanoma cells (1 × 10^6^ in 100 *μ*L/animal) to verify the effect of free curcumin (CUR) and CUR-loaded nanocapsules on melanoma tumor growth. Results showed that treatments significantly inhibited the tumor growth, 59.6% (1128.4 mm^3^ tumor volume) after treatment with free curcumin, 61.4% (1078.2 mm^3^ tumor volume) after treatment with Cur-loaded lipid nanocapsule suspensions, and 71.3% (801.4 mm^3^ tumor volume) after treatment with Cur-loaded polymeric nanocapsule suspensions, when compared to the control group treated with cell culture medium only (2791.0 mm^3^ tumor volume). Cisplatin was used as positive control and decreased 72.4% (770.0 mm^3^) of tumor volume [274].

Interleukin-2-loaded polymeric nanoparticle inhibited the tumor growth and can lengthen survival in mice B16F1-bearing melanoma. The antitumor effect was dose-dependent and the system demonstrated low toxicity, representing a new strategy in drug delivery system for melanoma gene therapy [234].

Cai et al., 2012, carried out a study to verify the influence of tumor-targeting nanocarrier in long-circulation effects promoted by PEGylated liposome. Results demonstrated that paclitaxel-loaded targeted PEGylated liposomes (TL-PTX) lengthen the half-life of paclitaxel 2.01-fold of conventional liposome and 3.40-fold of free paclitaxel in plasma. Higher accumulation of TL-PTX in tumor tissue, liver, and spleen was observed compared to conventional liposome and free paclitaxel [275].

Doxil, the first FDA-approved nanodrug [276], is corroborated to development of nanomedicine for melanoma therapy. After that, a lot of clinical trials have been done to verify the efficacy of nanodrugs to improve the survival and quality life in patients with melanoma, especially with poor prognosis [277].

In a clinical phase II study, Ugurel et al. (2004) verified that patients treated with liposomal doxorubicin as monotherapy present survival benefit. Outpatients setting (30 patients) were included on this study. Liposomal doxorubicin is used at 50 mg/m^2^ i.v. on days 1, 22, 43 and 64, subsequently at 40 mg/m^2^ at day 85 before first staging and in 4-week intervals thereafter. The results demonstrated that 7 patients stay alive more than 300 days and 5 patients more than 400 days [278].

Patients with cancer stage IV melanoma participated in an open-label, phase II study conducted by Hwu and coworkers (2006). Patients received a combination of 75 mg/m^2^ per day of temozolomide, during 6 weeks, there was a 2-week break between cycles, and they were continuously subcutaneously administrated PEGylated IFN-2b at 0.5 g/kg/week. Results showed that patient's median survival was 12 months and they were followed for 16 months and brain metastases were developed in any patients. Researchers concluded that a combination therapy promotes antitumor activity in metastatic melanoma [279].

## 5. New Approaches and Challenges

The wide range of compositions, morphologies, and particle sizes exhibited by drug delivery systems makes it difficult to understand their cellular uptake mechanisms. Thus, elucidating fundamental cellular processes that cells used to import and export select extracellular molecules may contribute to understanding the cellular internalization mechanisms of the systems and aid in selecting the appropriate system to transport active compounds [280]. Endocytosis of particles into cells depends not only on particle size, but also on surface coating and cell type [226, 227, 238].

Advances in nanotechnology based drug delivery systems have improved our understanding of the biological effects of nanotechnology-based systems, which will undoubtedly lead to important, clinically relevant improvements in drug delivery. New challenges in developing nanotechnology-based drug delivery systems for MEL antitumoral therapy include the feasibility of upscaling processes to quickly bring the innovative therapeutic techniques to the market and the potential for multifunctional systems that will fulfill several biological and therapeutic requirements, such as the system needing to be able to target tumor cells or tumor environment after systemic delivery. Further challenges include researches on efficiency of targeted anticancer therapies and imaging agents as well as international standards regarding their toxicology and biocompatibility.

So, the possibility of nanocarriers can promote the targeted cancer therapy and potentially early detection of cancer lesions; noninvasive imaging that permits determination of molecular signatures induces the concept of personalized medicine [281]. But not only that, the personalized medicine based on adjusted therapy to individual differences that can be detected by genetic test, guiding the choose of drug and their dosage. So, combining clinical and molecular biomarkers in nanomedicine contribute to improvement of the disease management [282].

## 6. Author's Opinion

Drug delivery systems represent an alternative strategy to carrier antineoplastic agents. Many advantages of drug delivery system have been described in recent studies such as better drug stability, better bioavailability, controlled drug release, long circulation time in blood, selective organs or tissue distribution, a reduction of the total dose required, and minimizing the toxic side effects.

Certain drug delivery characteristic can distinguish their application such as hydrogel that are stimuli-sensitive swelling-controlled release system. Liposome can encapsulate both hydrophobic and hydrophilic compounds. CD can form drug complexes and are biocompatible. LC protects the active ingredients from thermal degradation or photobleaching. SLN and NLC are maintained in the tumor for long period of the time due to low venous return and lymphatic drainage. PN forms reservoir-based drug delivery systems in nanocapsules and matrix organized polymeric chains in nanospheres. The wide range of compositions, morphologies, and particle sizes exhibited by drug delivery systems makes it variable mechanism for successful targeted delivery, while making it difficult to understand their cellular uptake mechanisms.

Another important aspect is identifying pathological systems' metabolism, tumor cell morphology, and microenvironment properties for accumulating the most drug delivery system at the site of action, at which decreased drug toxicity and adverse effects and biomarkers (antibodies, aptamers, peptides, and small molecules) can be identified, and molecules can be attached to the systems surface for successful targeted drug delivery to the site of action.

Thus, elucidating fundamental cellular processes that cells used to import and export select extracellular molecules may contribute to understanding the cellular internalization mechanisms of the systems and aid in selecting the appropriate system to transport active compounds. Endocytosis of particles into cells depends not only on particle size, but also on surface coating and cell type.

Several studies on cancer have been conducted worldwide, but peculiarities of tumor cells that distinguish them from normal cells are not completely elucidated, which made the delineation of targeted drug delivery for cancer therapy difficult.

Another problem is that chemotherapy drug delivery systems remain at an early development stage. Several studies have been executed to physicochemically characterize certain systems. However, the influence of systems to improve drug biological properties is understudied.

Tumor microenvironment plays an important role in tumorigenesis and may also influence the success rate of melanoma therapy. The drug delivery systems need to cross anatomical and physiological barriers of tumor microenvironment. However, many mysteries emphasize the complexity of the task.

In the recent decade, one of the most studied fields is nanotechnology-based drug delivery and various targeting mechanisms were discovered such as cancer-specific ligand for receptor-mediated active targeting (i.e., folate and hyaluronic acid); microenvironment-responsive molecules that respond to changes in pH, temperature, light, chemicals, and electromagnetic fields; PEGylation-induced passive targeting; electrostatics interaction and molecules that prevent the opsonization.

Drug delivery system for melanoma therapy may target the several pathways involved in melanoma development such as three-tiered Ras/Raf/MEK mitogen-activated protein kinase (MAPK); PI(3)K; NF-kappaB; p16INK4a/RB and ARF signalling pathways.

Although breakthrough in melanoma antitumor therapy research has been observed, more studies are necessary to better understand the role of drug delivery system in MEL therapy.

## Figures and Tables

**Table 1:** Particular nanocarrier structural components for improving drug targeting to the tumor tissue.

Components for successful targeted drug delivery in antitumor	Benefits in anticancer therapy	References
Active targeting		
Cholesterol	Cancer cells take up 100-fold more low density lipoprotein (LDL) than normal tissue due to upregulated LDL receptors in cancer cells for membrane synthesis during cell division associated with malignant transformation processes. Thus, LDL has been proposed as a drug carrier for anticancer agents.	[208, 239–245]
Polyunsaturated fatty acids (*α*-linolenic acid; linoleic acid; arachidonic acid; eicosapentaenoic acid; and docosahexaenoic acid).	They can be attached to the tumor cell membrane more easily, which results in disruption and fluidity of the cell membranes. Tumor progression is reduced by modulating p53, p16, and p27 expression and cell cycle regulation, as well as by inducing cell death by apoptosis and necrosis.	[246–248]
Hyaluronic acid	Hyaluronic acid is an extracellular matrix compound that specifically binds CD44, which is an extracellular membrane protein that regulates various cellular responses. CD44 is overexpressed in cancer cells, while normal cells underexpress this protein. Thus, CD44 is a good candidate biomarker for cancer cells.	[249–251]
Folic acid	Folate is important for producing and maintaining new cells because it can participate in nucleotide synthesis. Folates receptors are highly overexpressed in cancer cells. In addition, only the malignant cells, not normal cells, transport folate-conjugates; thus, the folate-drug conjugation can improve tumor-targeted drug delivery.	[248, 252–254]
		

Passive targeting		
Polysaccharides; polyacrylamide; polyvinyl alcohol; polyvinylpyrrolidone; PEG; PEG-containing copolymers (poloxamers; poloxamines; polysorbates; and PEG copolymer).	They prevent the opsonin binding to the nanoparticle surfaces and, consequently, recognition as well as phagocytosis of the nanoparticles by the mononuclear phagocytic system, which enhances the blood circulation time.	[212, 255–258]
Cationic surfactants	The positive charge of a cationic surfactant interacts through electrostatics with the negatively charged phospholipids that are preferentially expressed on the cancer cell surface.	[252, 259–261]

**Table 2:** Main advantages and disadvantages of each system.

Nanocarrier	Advantages	Disadvantages	References
Hydrogels	Cells and fragile drugs, like peptides, proteins, DNA, and oligonucleotides, could be protected by aqueous environment Good transport of nutrients to cells and products from cells Cell adhesion ligands easily modified them Can be injected as a liquid at body temperature; Usually biocompatible	Can be difficult to manufacture Usually mechanically weak Difficulty in encapsulating the drug Problems connecting with the cells Difficult to sterilize	[262]

Liposomes	They can be formed by natural or synthetic lipids Biodegradable Nontoxic Thermosensitive Hydrophilic and lipophilic molecules can be incorporated	High-energy sonication frequently causes oxidation and degradation of phospholipid Low-energy sonication requires long periods of sonication and can also be destructive to phospholipid High-pressure homogenization can confer decreased stability Application of volatile organic solvents	[263–266]

Micelles	Ease to prepare Good stability Many administration routes available	Risk of disintegration after administration	[267]

Cyclodextrins	Potential solubilizing and stabilizing agents Higher order complexes are possible Targeting water-insoluble drugs to the oral route	Some cyclodextrins have been shown to be irritants Safety concerns limited their use for parenteral administration	[268, 269]

Liquid crystals	They are easy to prepare Thermodynamically stable Composed of simple chemicals *In situ* phase transformations	Difficult to prepare and administer due to high viscosity Toxicity related to high surfactant concentration	[267, 270, 271]

Nanoparticles	They can be prepared by different methods Hydrophilic and lipophilic molecules can be incorporated They can change the surface Increased drug stability Possibility of controlled drug release and drug targeting	Toxicological assessment is uncompleted Low drug-loading capacities It is difficult to develop an analytical method for drug delivery Difficult to scale up the production Stability problems during storage	[195, 272]
